# The best laid plans of mice and women

**DOI:** 10.1371/journal.ppat.1006873

**Published:** 2018-04-12

**Authors:** Susan R. Ross

**Affiliations:** Department of Microbiology and Immunology, College of Medicine, University of Illinois at Chicago, Chicago, Illinois, United States of America; The Fox Chase Cancer Center, UNITED STATES

“The best laid schemes o' mice an' men

Gang aft agley”

From: To a Mouse, Robert Burns, 1785

My decision to become a scientist started with mice. In high school, after reading novels by C. P. Snow describing academic life at Cambridge University in England, I decided that I wanted to be a professor (little did I know that this vision of academic life was nothing like reality, at least in the United States). In sophomore year, my inner-city high school biology teacher taught us about the experiments of Jan Baptist van Helmont (1579–1644) showing that a piece of soiled cloth mixed with wheat yielded mouse pups after a 21-day incubation. When I asked why there was no mother, since mice are mammals and nurse their young, she replied, “Mice are rodents, not mammals.” I looked it up at the library, in those days without an online search engine, and found out that mice were indeed mammals. This sealed the deal—I wanted to be a biologist. While an undergrad, I was fortunate to find a summer research position in Bernard Goldstein’s lab at NYU. The Goldstein lab studied how air pollutants affected blood oxidation, and I was tasked with determining whether melanin conferred protection against ozone toxicity, using inbred mouse strains, including Himalayan mice. These mice have a thermolabile tyrosinase gene, so when raised in the cold, they have higher levels of melanin. My job was to take cold- and warm-exposed Himalayan mice and see if mice with more melanin were less susceptible to ozone toxicity (they weren’t).

Little did I know that this was the start of a career in mouse genetics. After my PhD work on adenovirus tumor antigens with Arnold Levine, I moved to do postdoctoral research in the new field of transcription in Keith Yamamoto’s lab. Keith’s lab used mouse mammary tumor virus (MMTV) as a “model gene” to understand glucocorticoid regulation of gene expression. MMTV is a milk-borne transmitted virus, but there are also endogenous, inherited copies of the retrovirus in the mouse genome, and this got me interested in the virus itself—why did it cause breast cancer in mice, and why were only some inbred strains of mice susceptible to infection? Why did mice retain endogenous viruses in their genome? I decided to make this the focus of my research.

At the time, the very new technique of transgenic mice was being pioneered, and it seemed that this might be useful for figuring out the virus’s biology. I was able to simultaneously accept a faculty position and to delay starting this position for a year while I did a short postdoctoral stint with Davor Solter at the Wistar Institute, learning mouse embryology and the technique of making transgenic mice. I was fortunate because an external fellowship from the Leukemia Society gave me the freedom to change postdoctoral mentors. More importantly, Davor was happy to let me work on my own project. This year of training was not only practical but exposed me to the mouse genetics community. Together with Davor, I published what I think was the first paper to study retrovirus biology using transgenic mice.

This began my journey of studying host–virus interactions using genetics. This journey has taken me from studies on the regulation of MMTV transcription to using mice to understand the genetics of susceptibility to infection to various viruses. For example, using transgenic mice, we showed that mice retain copies of endogenous MMTV in their genome as protection from exogenous infection. We also figured out why MMTV encoded a viral superantigen. Superantigens are presented by major histocompatibility class II proteins to CD4 T helper cells by interacting only with the T cell receptor (TCR) β chain, which causes massive stimulation of T cells. Tatyana Golovkina, then a postdoc in my lab, showed that transgenic mice expressing viral superantigens as endogenous genes were protected against infection. This is because superantigen-specific T cells are deleted during neonatal shaping of the immune repertoire, resulting in the loss of a reservoir of actively dividing, infection-competent cells. These studies showed the power of using genetically manipulated mice to study virus biology. However, while we know that there are endogenous retroviruses in all species, many of which play important roles in the biology of their hosts, it has not yet been demonstrated that these viruses directly protect against exogenous infection in humans, an area yet to be explored.

Since that time, we have gone on to identify many other genes that determine susceptibility to infection, including those involved in immune responses and virus entry. The identification of entry receptors has also helped us understand species-specific virus tropism, including why MMTV likely doesn’t infect humans. Through these studies, we have expanded our virus portfolio to include both human and mouse retroviruses as well as New World hemorrhagic fever arenaviruses, and we have gone into research areas I never thought I would be involved in, including drug discovery and recently even neurology. While my lab now uses a variety of approaches to identify the genes that confer resistance and susceptibility, we always end up in the mouse. The mouse has sometimes led us from our best-laid plans, but more often than not, it has led us to new, exciting avenues of exploration.

**Image 1:**
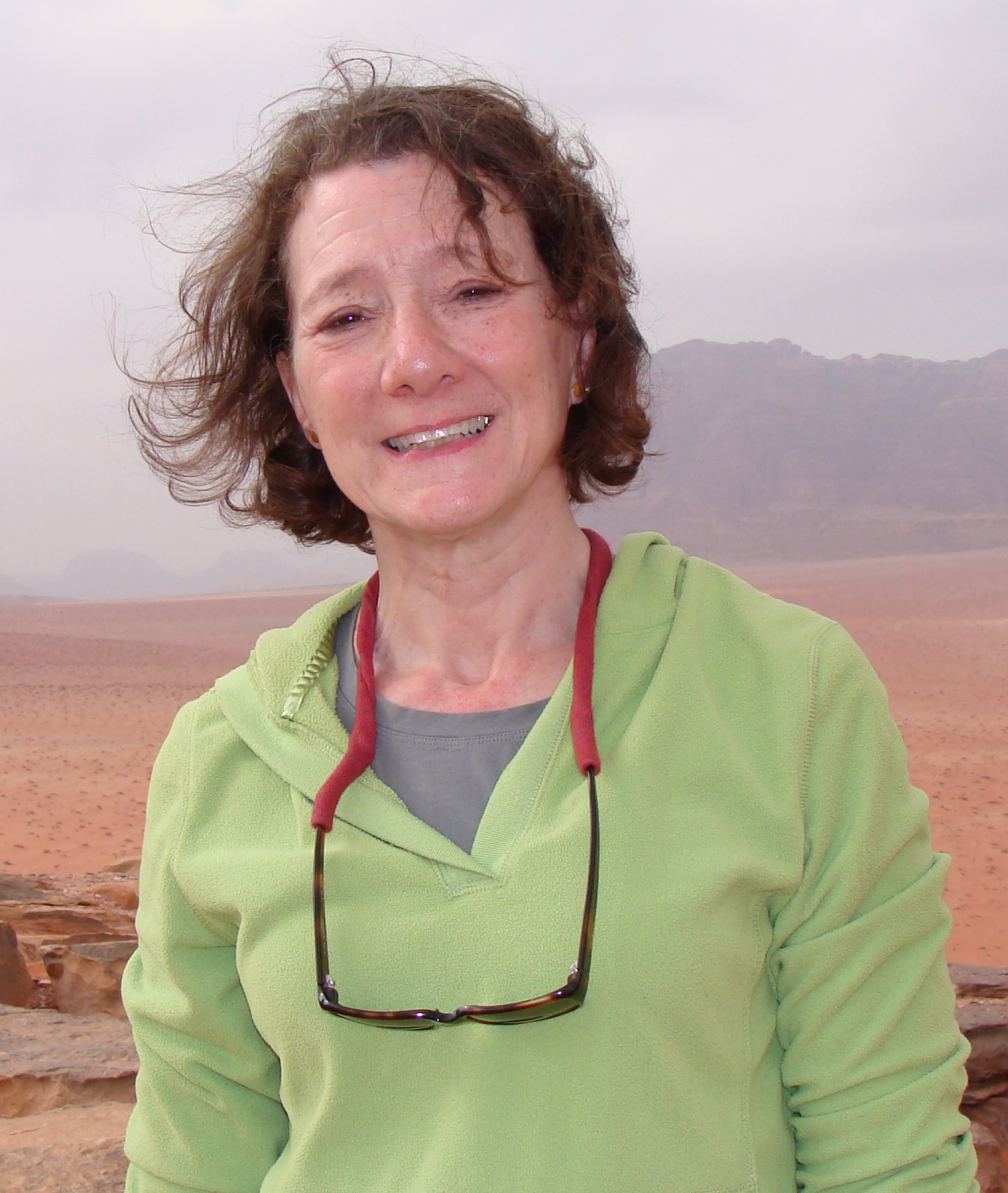
Susan R. Ross.

